# The Quality Assessment of Commercial Lycium Berries Using LC-ESI-MS/MS and Chemometrics

**DOI:** 10.3390/plants8120604

**Published:** 2019-12-13

**Authors:** Mariam Jarouche, Harsha Suresh, James Hennell, Shaun Sullivan, Samiuela Lee, Swastika Singh, Declan Power, Cindy Xu, Cheang Khoo

**Affiliations:** 1 Herbal Analysis and Pharmacological Laboratories (HAPL), National Institute of Complementary Medicine (NICM), Western Sydney University, Campbelltown, NSW 2560, Australia; mjarouchee@gmail.com (M.J.); 15748036@student.westernsydney.edu.au (J.H.); 16720969@student.westernsydney.edu.au (S.S.); swastika.singh@westernsydney.edu.au (S.S.); 2 School of Medicine, Western Sydney University, Campbelltown, NSW 2560, Australia; 3 Reference Standards Department, National Measurement Institute (NMI), North Ryde, NSW 2113, Australia; sam.lee@measurement.gov.au; 4 Wentworth Institute, Surry Hills, NSW 2010, Australia; cindy@win.edu.au (C.X.); khoo2031@gmail.com (C.K.)

**Keywords:** *Lycium*, Herb MaRS, LC-MS, Chemometrics, PCA

## Abstract

*Lycium* (also known as Goji berry) is used in traditional Chinese medicine (TCM) with claimed benefits, including eye and liver protection, immune system fortification and blood glucose control. The commercially available product comes from either the *L.* *barbarum* or *L. chinense* species, with the former dominating the marketplace due to its better taste profile. The main objective of this study was to develop a validated LC-ESI-MS/MS method to quantify multiple key bio-active analytes in commercially available *Lycium* berries and to qualitatively assess these samples using a principal component analysis (PCA). A LC-ESI-MS/MS method for the quantitation of seven analytes selected using the Herbal Chemical Marker Ranking System (Herb MaRS) was developed. The Herb MaRS ranking system considered bioavailability, bioactivity and physiological action of each target analyte, its intended use and the commercial availability of an analytical standard. After method optimization combining high resolving power with selective detection, seven analytes were quantified and the *Lycium* samples were quantitatively profiled. Chromatographic spectra were also obtained using longer run-time LC-UV and GC-MS methods in order to qualitatively assess the samples using a principal component analysis (PCA). The result of the method validation procedure was a 15.5 min LC-ESI-MS/MS method developed for the quantification of seven analytes in commercial *Lycium* samples. Wide variation in analyte concentration was observed with the following results (analyte range in mg/g): rutin, 16.1–49.2; narcissin, 0.37–1.65; nictoflorin, 0.26–0.78; coumaric acid, 6.84–12.2; scopoletin, 0.33–2.61; caffeic acid, 0.08–0.32; chlorogenic acid, 1.1–9.12. The quantitative results for the *L. barbarum* and *L. chinense* species samples indicate that they cannot be differentiated based on the bio-actives tested. A qualitative assessment using PCA generated from un-targeted LC-UV and GC-MS phytochemical spectra led to the same conclusion. The un-targeted quantitative and qualitative phytochemical profiling indicates that commercial *L. barbarum* and *L. chinense* cannot be distinguished using chemical analytical methods. Genetic fingerprinting and pharmacological testing may be needed to ensure the efficacy of commercial *Lycium* in order to validate label claims.

## 1. Introduction

The *Lycium* genus comprises of approximately 75 species and is the most widely distributed *Solanaceae* genera native to arid and sub-arid regions of South America, Southern Africa, Eurasia and Australia [1]. It grows primarily in northwest China and Tibet [2]. The fruit is shade dried until the skin shrinks then sun dried until the outer skin becomes dry and hard but with the pulp still soft. China is the main supplier of *Lycium barbarum* berry products, with 95,000 tons of exports totaling $120 million in 2004 [3]. Effective marketing has led to the product being perceived as a “super food”.

Goji is the common name for the berry of *L. barbarum* and *L. chinense,* which is sold as a food and dietary supplement. The berry is shade dried until it shrinks, then sun dried until the outer skin hardens but with the pulp still soft. Generally, the *L. chinense* species is grown in southern China while the *L. barbarum* is grown in the north where the plant is somewhat taller but there is not always a clear distinction in the morphology of their fruits as there is intra-species variation. A review paper on the subject states that “The fruits of *Lycium* species possess a highly similar anatomy and tissue structure and differentiation based on morphological and histological analyses is very delicate [4].” Additionally, the chemical composition is influenced by factors such as natural variation, growth conditions (such as soil type and climate), time of harvest and by post-harvest treatment, including storage conditions. While a botanically certified sample of *L. barbarum* fruit is commercially available, the authors are unable to source one for the *L. chinense* species. While there is a broad view that these two species are similar, they may have different applications in traditional Chinese medicine (TCM). For reasons that are not clear, it appears that the fruit of the *L. barbarum* is more often used while the roots and leaves of the *L. chinense* are favored. There is a study that reported that the leaf extract of *L. chinense* had more polyphenols, flavonoidic compounds and caffeic acid derivatives than that from *L. barbarum* as well as showing greater microbial activity. The fruits of the *L. barbarum* and *L. chinense* varieties were not examined concurrently in this study [5]. Another study reported a difference in the phytochemical composition of the fruit where that of the *L. barbarum* has a higher sugar content than *L. chinense* and that soil chemistry greatly affects the concentration of sugar [6]. A study comparing the taste pattern (using taste sensors) and betaine (an amino acid) concentration reported that the *L. barbarum* had double the sugar and about 15% more betaine than the *L. chinense*. The samples in the study were obtained from 15 distributors, with the variability in the concentrations being surprisingly small at about 20% and 17% RSD (relative standard deviation) for betaine and sugar respectively [7]. There is a view that the only reliable way to distinguish between the two species is via molecular techniques such as random amplified polymorphic DNA analyses [8,9]. Most of the studies on the phytochemical differences between the two *Lycium* species obtain their *L. chinense* samples directly from the geographic region from which it is known to grow it rather than from certified botanical samples. 

Un-targeted chromatographic profiling analysis has used to obtain chromatographic profiles for *L. barbarum* and *L. chinense* samples by determining all detectable constituents without necessarily identifying or quantifying a specific compound in a single analysis [10,11]. The more recent research regarding *Lycium* has focused on either the anti-oxidant or anti-microbial aspect [5,12,13] with LC-UV methodologies [14,15]. LC-UV (or PDA) detects substances with a chromophore (UV absorbing part of the molecule) while GC-MS detects volatile substances. Both are useful for un-targeted analysis as they provide rich chromatographic spectra and detect substantially different groups of substances. Targeted analysis seeks to detect and quantify a specific number of selected chemicals and as such, is not as spectrally rich as the two previous techniques [16]. 

LC-ESI-MS/MS is more suited for targeted analysis in a complex matrix due to being sensitive and selective. When there is a greater possibly of chromatographic peak overlap and misidentification, the MS detector can minimize this problem by providing peak identity confirmation. The MS can be set to detect only a specific mass-to-charge ratio (*m*/*z*) and if tandem MS/MS is used, the precursor *m*/*z* can be subjected to fragmentation by a collision gas and the product *m*/*z*’s detected with their relative ratios compared between the standard and samples. Furthermore, analytes without a chromophore can be detected using MS/MS. 

The main aim of this study was the development and validation of a rapid UPLC-MS (ultra-performance liquid chromatography with mass spectrometry detection) method to determine seven major bio-actives in *L. barbarum* and *L. chinense.* The validated method can be used for better quality control (QC) in commercial *Lycium*, thereby enabling some measure of standardisation, providing the consumer with a more consistent product [17]. A secondary objective was to examine if *Lycium* species in the marketplace labelled as ‘*L. barbarum’* and ‘*L. chinense’* are qualitatively different in principal component analysis (PCA) using spectra from LC-UV and GC-MS. The target analytes were selected using the Herbal Chemical Marker Ranking System (Herb MaRS) developed at the National Institute of Complementary Medicine (NICM) to assess the bioactivity, physiological activity and the bioavailability of each bio-active analyte present in any herb or herbal formulation [18]. The chemical structures of seven target bio-active analytes are shown in Table 1 and their pharmacological activities and rankings are shown in Table 2. 

## 2. Methods

### 2.1. Instrumentation 

A Waters ACQUITY UPLC system (Milford, MA, USA) consisting of a binary sample manager, a sample manager, including the column heater, detector and sample organiser coupled to a Xevo TQ tandem-quadrupole mass spectrometer equipped with a Z-spray electrospray interface, was used. The binary solvent manager used two individual serial flow pumps to deliver a parallel binary gradient mixed under high pressure with built-in solvent degassing and up to four solvent select valves. The ACQUITY system is capable of pumping mobile phase at pressures up to 15,000 psi. Negative electrospray ionization (ESI) was performed in the multiple reaction monitoring (MRM) mode. The loop size was 10 µL. Separation was carried out on an ACQUITY UPLC BEH C18 column (100 mm × 2.1 mm, 1.7 µm). 

The herb grinder used for processing raw samples was a M20 Universal Mill IKA® instrument (Werke Staufen, Germany). The ultrasonic bath was a Branson 1510 from Branson Ultrasonics (Danbury, CT, USA), and the centrifuge used was a Beckmann GP from Beckmann Coulter (Brea, CA, USA). An Adam AFA-210LC analytical balance (Oxford, CT, USA) and a Sartorius SE-2 micro analytical balance (Gottingen, Germany) were used to weigh the samples and analytical standards. 

### 2.2. Reagents, Chemicals and Samples

The analytical reference standards rutin (94%), caffeic acid (99%), coumaric acid (98%), chlorogenic acid (95%), and scopoletin (99%) were purchased from Sigma-Aldrich (St Louis, MO, USA); nictoflorin and narcissin were of secondary grade from Phytomarker Ltd. (Tianjin, China). The primary grade standards have purity and spectroscopic standardisation while the secondary grade standards have purity by LC-UV only. The calibration curves were prepared with adjustment for standard purity. 

UPLC grade methanol and AR grade ethyl acetate was obtained from Biolab (Clayton, VIC, Australia). AR grade formic acid was obtained from Univar (Downers Grove, IL, USA). Phosphorus pentoxide (used as desiccant) was from Sigma-Aldrich (St Louis, MO, USA). Air, argon, helium, hydrogen and nitrogen were of ultra-high purity grade from Coregas (Yennora, NSW, Australia). Purified water (>18 MΩ cm) was obtained from an Elga Purelab Prima and Purelab Ultra high purity water system (Aubervilliers, France).

Twelve samples of the dried raw herb berries (8 labelled as *L. barbarum* and 4 as *L. chinense*) were obtained from local retail shops in Sydney, Australia. The authenticity of the raw material was established by chromatographic profile comparison against a certified reference sample of *L. barbarum* berry (batch number AAT15209CRB) purchased from Alkemists Pharmaceuticals (Stanton, CA, USA). A certified reference sample of *L. chinense* berry was not commercially available. The certified *Lycium* sample was primarily used for comparison to the commercial *Lycium* samples in the PCA of LC-UV and GC-MS spectra. Analytical method validation was performed on the *L. barbarum* sample LB7.

### 2.3. Sample Extraction and LC Mobile Phase Preparation

The raw samples were dried over phosphorus pentoxide in a desiccator for one week, then ground to pass through a ≤ 250 μm sieve before being stored in the desiccator under vacuum. To determine the optimal extraction solvent, various aqueous methanolic solutions were investigated and the solvent that resulted in the largest overall peak area was subsequently selected for use. The concentration of the analytes in the unspiked samples was determined by weighing a known amount of the ground raw herb (~1.0 g) into a 10 mL volumetric flask and extracted by sonication in 7 mL 50% aqueous methanol for 2 × 30 min, (15 min cooling interval between sonications). After cooling, the flask was made up to volume with the extraction solvent and mixed by vortexing. The supernatant was then transferred to a centrifuge tube and centrifuged at 4000 rpm for 10 min. The aliquot of the supernatant was then filtered through a 0.22 μm PVDF membrane filter into a 2 mL auto sampler vial for analysis. The filtrate was stored at 4 °C if not analysed on the same day. Each sample was analysed in seven replicates (extraction and analysis). The mobile phase program is shown in Table 3, with a flow rate of 0.2 mL/min and a run time of 15.5 min. 

### 2.4. Preparation of Stock Calibration Solution Using Analytical Standards

Individual solutions of 5000 μg/mL of rutin, chlorogenic acid, coumaric acid were prepared by weighing 50.0 mg of analytical standard (using a microbalance) into a 10 mL volumetric flask and adding approximately 7 mL of methanol before sonication for 5 min or until the solid was dissolved. The solution was then cooled before making up to volume with methanol. In a similar manner, individual solutions containing 500 μg/mL of narcissin, nictoflorin, scopoletin and caffeic acid were prepared from 5.0 mg of analytical standard. The solutions were stored at 4 °C and discarded if not used within 2 weeks. 

A mixed standard stock solution containing 1140 μg/mL rutin, 39.6 µg/mL chlorogenic acid, 89.4 μg/mL coumaric acid, 60 μg/mL narcissin, 11.6 μg/mL nictoflorin, 17.4 µg/mL scopoletin and 1.64 μg/mL caffeic acid was prepared by adding 5.7, 1.7, 3.7, 2.5, 0.50, 0.80 and 0.10, mL of the respective individual analytical standards into a 25 mL volumetric flask and making up to volume with methanol. This mixed standard stock solution was used as the spiking solution for analyte recovery studies and preparation of working calibration solutions. 

The working calibration solutions were prepared by diluting 50, 150, 250, 500 and 1000 μL of the mixed standard stock solution to 1000 µL with methanol, representing a 1/20, 1/10, 1/4, 1/2 and the undiluted standard respectively. The linear range for each analyte is the linear calibration range for the standards in the calibration solution. This encompasses the concentration range for each analyte of interest in every commercially purchased *Lycium* sample. 

### 2.5. Recovery Studies 

To determine the analyte extraction efficiency of the method, accurately weighed 1.0 g of the ground herb sample was transferred into 10 mL volumetric flasks and 0.5 mL of the spiking stock solution was added for the 100% spike level. For the 50% and 200% spike levels, the amounts were adjusted in proportion. The concentration of the mixed spiking solution was arranged such that for the 100% spike level, the resultant peak area would be twice that of the unspiked sample. Seven replicates were used for each spike level to obtain a total of twenty-one injections for the three spike levels. The spiking solvent was evaporated overnight in a fume hood.

### 2.6. LC-ESI-MS/MS Conditions 

The ESI source conditions were set with nitrogen desolvation gas at 800 L/h heated to 350 °C and argon used as the collision induced dissociation gas at 0.15 mL/min. The collision gas pressure was maintained at 4.3 × 10^−6^ bar in the collision cell. The scan time was set at 0.005 s, the extractor cone was set at 3V and cone gas flow set at 20 L/h. The source block temperature was set at 150 °C, the capillary voltage in the negative (−) ESI mode was 2.40 kV. Manual tuning for optimal cone voltage and collision energy was performed using the built-in instrument fluidics system. Dwell times for the multiple reaction monitoring (MRM) channels were automatically calculated by the software, the required data points were selected for peak determination. The inter-channel and inter-scan delay time was set to 3 milli-seconds. 

System operation and data acquisition were controlled using the Waters Mass Lynx 4.1 software. Two or if possible three MRM products (or transition *m/z*’s) were chosen for each target analyte, with the most abundant product used as the quantifier and the others used as the qualifiers. Two product ions with matching intensities between the standard and sample peaks meet the analyte identity confirmation standard set by the European Commission Directorate for Agriculture guidelines [43]. The ESI polarity, precursor and product ions were monitored, and the argon collision voltages required to achieve the transitions and the dwell times used are summarised in Table 4.

### 2.7. LC-UV and GC-MS Methods Used to Obtain Spectra for PCA Analysis

The LC-UV spectral profiles used to study the phytochemical variability of the *L. barbarum* and *L. chinense* samples using principal component analysis (PCA) were generated. Analysis was performed using a Varian (California, USA) Prostar solvent delivery system comprising of 2 × 210 single pumps equipped with a 335-photodiode array detector (PDA), a ‘9×0 mm’ analytic flow cell (Varian Inc., Australia), column valve module 500 and 430 auto sampler injector. Solvents were degassed using a model AF DG2 in-line degasser (Waters, USA). The system was controlled using Varian Star Workstation version 6.20. A security C18 guard column (3 × 4.6 mm, 5 μm) was used with a Phenomenex Luna C18 column (150 × 4.6 mm, 5 µm) (Torrance, CA, USA) analytical column. The injection volume was 10 µL and column temperature 30 °C. The PDA was set to acquire data from 200–400 nm and the chromatograms visualized at 280 nm. The mobile phase was acetonitrile (A) and water containing 0.1% formic acid (B) with a flow rate of 1 mL/min. Each sample was injected in triplicate and the run time 100 min. Initially 95% mobile phase B was held for 5 min. A slow gradient was applied to 55 min where the mobile phase B was 90%. Another gradient was applied until 80 min where mobile phase B was 80%. Mobile phase B was changed to 5% at 81 min and then held at 5% until 90 min. Then mobile phase B was set to 95% at 91 min and held to 100 min as the wash phase.

GC-MS was also used to obtain chromatographic spectral profiles for PCA analysis, and the method used is described as follows. The injection volume programmed into the auto sampler was 1 μL, pre-cleaning the syringe with ethanol once and the sample five times before each injection. The syringe was rinsed with ethanol five times after each injection. The injector temperature was set at 200 °C using a split ratio of 10:1. The column pressure was programmed to maintain a constant flow of 1.5 mL/min. The initial oven temperature was 50 °C held for 1 min, then increased at a rate of 10 °C/min to 300 °C and held for 3 min. The oven temperature was then increased to 350 °C for 2 min after each run to clean the column. The MS transfer line was maintained at 250 °C, the EI source at 230 °C and the quadrupole at 150 °C. The helium carrier gas was set to flow at 1.0 mL/min. The MS scan range was 40–500 *m/z*. Each run was 140 min and analysis performed in triplicate.

### 2.8. PCA Analysis

The LC-UV and GC-MS spectral data sets of the *Lycium* samples were subjected to principal component analysis (PCA) which can help identify components that explain the variability in a data set. LC-UV and GC-MS spectra were preferred over LC-ESI-MS/MS data for PCA since they are more spectrally rich and provide wider context information regarding qualitative information. In the case of LC-UV, this was the chromatographic spectra at 280 nm (which includes multiple compounds with chromophores not quantified in the LC-ESI-MS/MS method). The GC-MS spectra were used to study the variability of volatile compounds in *Lycium*. Using the PCA from both these analytical methods, it could be ascertained whether the *L. barbarum* and *L. chinense* samples separate into two distinct groups in PCA analysis. 

Data processing and statistical analysis was performed using the “R” statistical computing package [44] and the add-on package “msProcess” [45]. This enabled chromatogram modification by removal of instrumental noise, peak retention time variation between samples, baseline drift, and identification of peaks thereby minimising sample variation due to instrumental factors. Extract chromatograms were pre-processed, peaks were identified in each chromatogram and then aligned to remove small variations in peak retention times between chromatograms. The chromatograms were then normalized and binned. Whenever a peak was detected in any chromatogram, the amplitude at that retention time was measured across all chromatograms in order to build a matrix of peak amplitudes. One the dataset was constructed it was used to generate a “R.data” file which was then used to create the PCA plots for the LC-UV and GC-MS chromatographic spectra. 

## 3. Results and Discussion

### 3.1. Chromatographic Data and Recoveries

**Figure 1:**
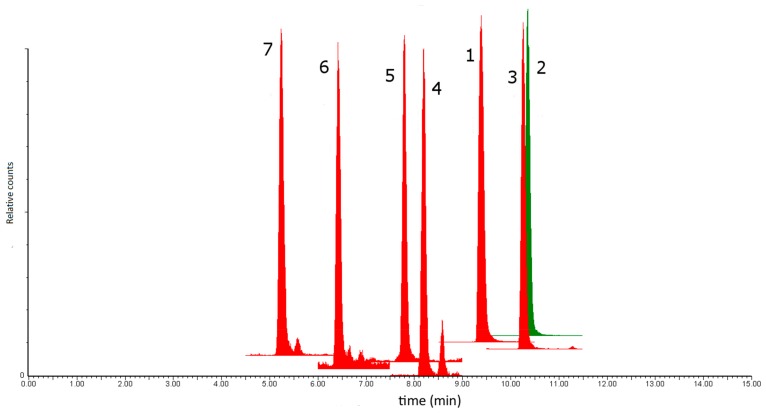
Representative LC-ESI-MS/MS chromatogram of the extract of sample LB7 containing (1) Rutin, (2) Narcissin, (3) Nictoflorin, (4) Coumaric acid, (5) Scopoletin, (6) Caffeic acid and (7) Chlorogenic acid.

A representative MS chromatogram for the method validation sample LB7 is shown in Figure 1. Good recoveries (90.8%–109.5%) were obtained for the analytes of interest at all the spiking levels as shown in Table 5. The 50% spike level showed slightly lower recoveries than the 100% and 200% spiking levels. This is likely due to constant loss of the analyte due to the absorption onto glassware surfaces. The average recovery RSD values are <10% for all the analytes.

### 3.2. Precision and MS Identity Confirmation

The analyte precisions of quantitation are shown in Table 6. The precision data shown in the table is the overall method precision of *n* = 7 extractions of validation sample LB7 with each extraction injected in triplicate. The two main contributors to method precision are the instrumental SD and the extraction process SD, which primarily arises from errors in weighing, transferring and volume adjustment. The method precision encompasses both the instrumental and extraction process SD and if the two main sources of error were monitored independently, the instrumental error contributes approximately one third to overall SD. The MS identity confirmation data is shown in Table 7. Most results are well within the tolerances described by the guidelines set out in the European Commission Directorate for Agricultural guidelines [43] except for *m*/*z* 93 of chlorogenic acid where identity confirmation fails by 1% where the required tolerance is ± 25% but ± 26% was obtained. Identity confirmation also fails for caffeic acid which had only *m/z* 135 product ion for identity confirmation where two ions are required. The calibration curves for all the analytes show good linearity with R^2^ > 0.999.

### 3.3. Analyte Concentrations and Fold Variation

The concentrations of the monitored analytes in twelve *Lycium* samples are shown in Table 8. The seven bio-actives are present in both the *L. barbarum* and *L. chinense* samples except for caffeic acid in the *L. barbarum* samples LB2 and LB4 and chlorogenic acid in LB2. High concentration components such as rutin and coumaric acid show low fold variation at 3.1 and 1.8 respectively. Scopoletin had the highest fold variation at 7.8 but this is due to its low concentration across the samples. This shows that the *L. barbarum* and *L. chinense* samples are not chemically divergent, at least in the composition of major bio-actives that have a role in their known pharmacological activity. Though the variation in chemical composition is significant, it is lower than many other TCM herbs reported.

### 3.4. PCA Analysis

PCA analysis of *Lycium* was performed using LC-UV chromatograms visualized at 280 nm. At this wavelength, a satisfactory number of peaks is observed—at shorter wavelengths, many peaks are observed because most substances absorb in the short UV (<220 nm) whereas at long wavelengths (>350 nm), the chromatogram can be relatively featureless. LC-UV can detect volatile and non-volatile compounds with a chromophore while GC-MS monitors the volatile compounds. The *y*-axis is the Euclidean distance for principal component PC1, and the *x*-axis is the distance for the principal component PC2. The closer the samples are in terms of Euclidean distance, the more similar they are to each other. Since two different chemical monitoring techniques are used for comparison, the qualitative phytochemical variability of *Lycium* is understood in greater depth. 

**Figure 2:**
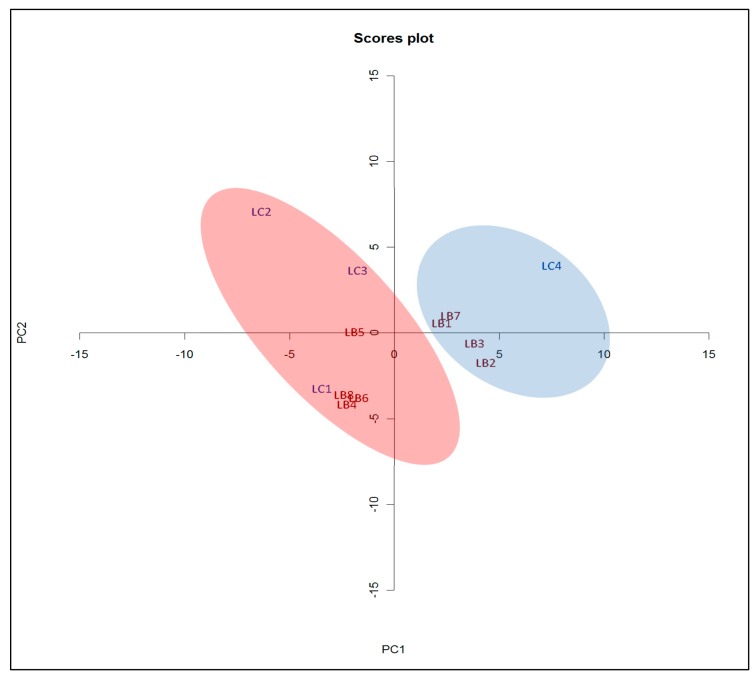
LC-UV spectra PCA score plot for the *L. barbarum* and *L. chinense* samples.

The chromatograms are visually similar, suggesting that there is little chemical difference between the *L. barbarum* and *L. chinense* samples at least among the monitored bio-actives. The LC-UV PCA score plot in Figure 2 shows the *L. barbarum* and *L. chinense* samples grouped into two close clusters with significant overlap indicating that their profiles are near indistinguishable.

**Figure 3:**
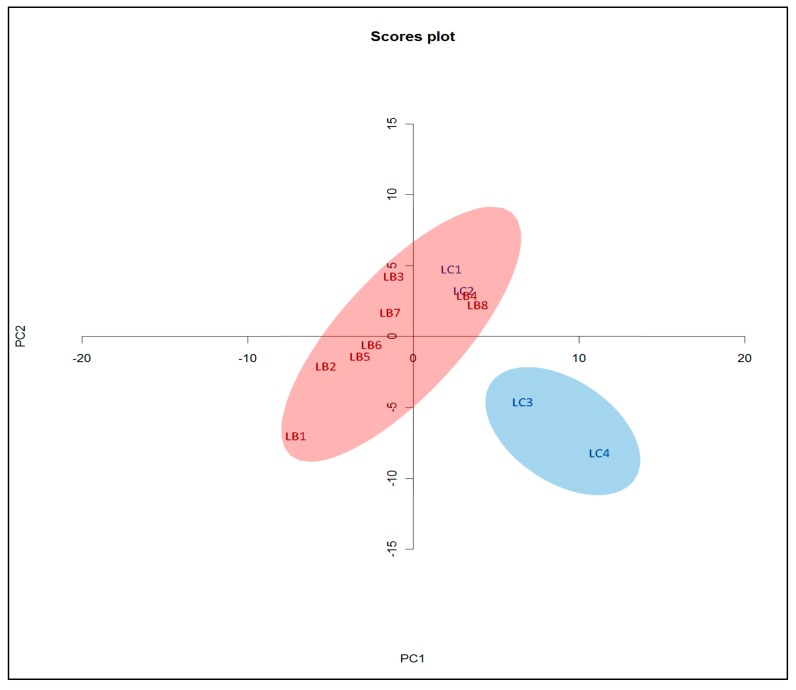
GC-MS spectra PCA score plot for the *L. barbarum* and *L. chinense* samples.

As with the LC-UV chromatograms, the GC-MS chromatograms are visually similar for the *L. barbarum* and *L. chinense* samples. The PCA score plot of the GC-MS chromatograms is shown in Figure 3. The LC3 and LC4 samples are shown to be part of an outlier group. The presence of distinct un-analyzed volatile compounds in these samples could be the reason they are appear as outliers.

Based on the GC-MS PCA score plot, the LC1 and LC2 *L. chinense* samples more closely resemble those of *L. barbarum* while the remaining *L. chinense* samples are phytochemically indistinguishable. Outliers LC3 and LC4 could be due to natural variation and/or growth conditions and post-harvest treatment. Nevertheless, the seven monitored bio-actives are still present in LC3 and LC4. 

## 4. Conclusions

A simple and rapid 15.5 min LC-ESI-MS/MS method was developed and validated to monitor seven bio-actives in *Lycium* samples of the *barbarum* and *chinense* varieties. Total concentration of the monitored bio-actives could be used to describe herbal quality. It is probable that these two *Lycium* berries, though labelled differently, are essentially the same and likely to give similar physiological effects. Any variation in chemical composition likely arises from factors described previously rather than species type. While there are archetypal *L. barbarum* and *L. chinense* plants there may be hybrids, further blurring their distinction. Mislabeling is another consideration, though the minor price difference between the two *Lycium’s* makes this occurrence more likely accidental than deliberate. 

## Figures and Tables

**Table 1:** Structures of the seven target analytes monitored in *Lycium*.

Compound	Chemical Structure
Rutin	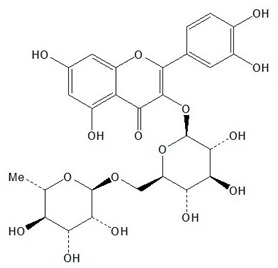
Narcissin	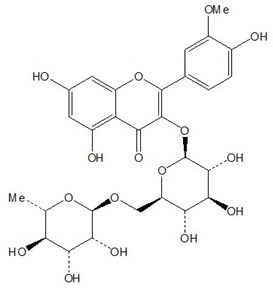
Nictoflorin	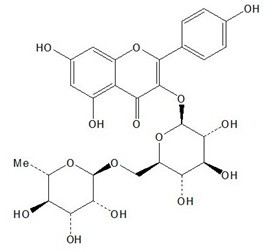
Coumaric acid	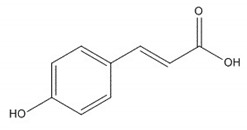
Scopoletin	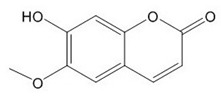
Caffeic acid	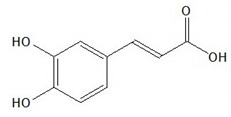
Chlorogenic acid	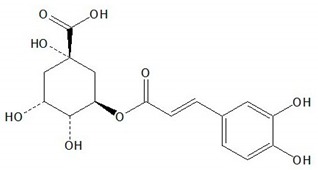

**Table 2:** Reported pharmacological activities of target analytes in *Lycium.*

Analyte [References]	Reported Activity	Herb Mars Ranking ^a,b^
Rutin [3,19,20,21,22,23]	Anti-hepatotoxic, anti-oxidant, cAMP-phosphodiesterase-inhibitor, vasopressor, vasodilator, anti-inflammatory, cytoprotective	5
Narcissin [24,25,26,27]	Anti-inflammatory, anti-oxidant, hepatoprotective	4
Nictoflorin [28,29,30,31,32]	Anti-hepatotoxic, anti-oxidant, iNOS-Inhibitor, cAMP-phosphodiesterase-inhibitor, TNF-alpha-inhibitor, neuroprotective	4
Coumaric acid [33,34]	Anti-hepatotoxic, anti-oxidant	3
Scopoletin [35,36,37]	Anti-hepatotoxic, anti-oxidant	3
Caffeic acid [38,39,40]	Anti-oxidant, anti-inflammatory, anti-hepatotoxic, hepatotropic	2
Chlorogenic acid [41,42]	Hepatotropic, anti-inflammatory	2

^a^: Herbal Chemical Marker Ranking System (5). ^b^: The ranking score ranges from 0 to 5, with 0 being the least and 5 being the most suitable.

**Table 3:** Mobile phase gradient program for the LC-ESI-MS/MS method.

Time (min)	Water (with 0.1 % *v/v* Formic Acid) %	Acetonitrile %
Initial	80	20
1.0	75	25
6.0	55	45
11.0	35	65
13.0	25	75
15.0	80	20
15.5	80	20

**Table 4:** LC-ESI-MS/MS monitoring conditions.

Analyte	ESI Polarity	Precursor *m/z*	Product *m/z*	Respective Voltages (V)	Dwell Time (s)
Rutin	-	[M-H]^−^ = 609	255, 271, 300	50, 62, 40	0.016
Narcissin	-	[M-H]^−^ = 623	299, 315	48, 30	0.017
Nictoflorin	-	[M-H]^−^ = 593	255, 284	54, 34	0.017
Coumaric acid	-	[M-H]^−^ = 164	93, 120	12, 26	0.016
Scopoletin	-	[M-H]^−^ = 191	103, 176	24, 16	0.016
Caffeic acid *	-	[M-H]^−^ = 179	135	15	0.195
Chlorogenic acid	-	[M-H]^−^ = 353	93, 191	50, 50	0.095

*: Only one product *m/z* ion was observed for caffeic acid.

**Table 5:** Analyte recoveries.

Analyte	Spike Levels ^a,c,d^						Cumulative Results	
	50%		100%		200%			
	Recovery %	RSD %	Recovery %	RSD %	Recovery %	RSD %	Average Recovery ^b^ %	RSD %
Rutin	93.1	3.7	90.2	7.9	89.1	8.6	90.8	6.7
Narcissin	105.6	3.4	101.7	1.7	100.5	2.5	102.6	2.8
Nictoflorin	92.2	3.8	94.5	2.9	95.2	2.5	93.9	3.1
Coumaric acid	98.4	7.5	88.6	9.3	102.0	3.8	96.3	6.8
Scopoletin	89.6	7.0	95.3	5.5	91.2	3.3	92.0	5.3
Caffeic acid	89.9	7.1	110.8	9.5	113.7	4.2	104.8	1.9
Chlorogenic acid	101	9.2	112.8	8.2	114.3	7.4	109.4	4.5

^a^: % Recovery ± % RSD calculated from *n* = 7 extractions and injected in triplicate. ^b^: Average recovery of all three spiking levels ± % RSD. ^c^: LOD was 0.005 mg/g. ^d^: LOD was 0.003 mg/g.

**Table 6:** Precision of quantitation.

Analyte	Linearity (R^2^)	Linear Range (µg/mL)	Precision ^a^		LOD (mg/g) ^b^	LOQ (mg/g) ^c^
			Amount (mg/g) (± % RSD)	RT (min) (± % RSD)		
Rutin	0.9992	57.0-1140.0	34.8 (4.3)	9.37 (0.02)	0.42	1.4
Narcissin	0.9995	3.0-60.0	1.65 (2.8)	10.30 (0.01)	0.13	0.4
Nictoflorin	0.9994	0.6-11.6	0.77 (5.9)	10.20 (0.01)	0.14	0.47
Coumaric acid	0.9991	4.5-89.4	10.3 (6.9)	8.30 (0.02)	0.25	0.84
Scopoletin	0.9995	0.9-17.4	1.68 (4.3)	7.75 (0.03)	0.06	0.20
Caffeic acid	0.9995	0.1-1.6	0.11 (9.3)	6.35 (0.01)	0.04	0.12
Chlorogenic acid	0.9991	2.0-39.6	4.19 (5.9)	5.21 (0.01)	0.74	2.47

^a^: Average and RSD calculated from *n* = 7 extraction replicates injected in triplicate. ^b^: Limit of detection (LOD) is three times the standard deviation (SD) for each analyte in LB7. ^c^: Limit of detection (LOQ) is ten times the standard deviation (SD) for each analyte in LB7.

**Table 7:** Identity confirmation of the analytes.

Analyte	Relative Intensity				Tolerances	
	*m/z*	Standard	Sample	Relative Difference (± %) ^a^	Permitted Tolerance (± %) ^b^	Pass/Fail
Rutin	300	100	100	-	20	Pass
	271	61	60	1.6	20	Pass
	255	31	30	3.2	25	Pass
Narcissin	315	100	100	-	20	Pass
	299	61	53	14	20	Pass
Nictoflorin	284	100	100	-	25	Pass
	255	86	73	16	25	Pass
Coumaric acid	120	100	100	-	25	Pass
	93	29	28	3.5	25	Pass
Scopoletin	176	100	100	-	25	Pass
	103	40	36	10	25	Pass
Caffeic acid	135	100	100	-	25	Pass
Chlorogenic acid	191	100	100	-	25	Pass
	93	50	37	26	25	Fail

^a^: Relative difference = [Intensity of sample—intensity of pure standard)/(intensity of pure standard)] × 100. ^b^: Maximum permitted tolerance from the European Commission Directorate for Agricultural guidelines [43].

**Table 8:** Concentrations of target analytes.

Analyte Concentrations in Sample (mg/g) (± % RSD) ^a^								
Sample ^d^	Rutin	Narcissin	Nictoflorin	Coumaric Acid	Scopoletin	Caffeic Acid	Chlorogenic Acid	Total Concentration
LB1	16.1 (9.3)	0.37 (3.0)	0.37 (8.5)	6.84 (4.5)	0.77 (6.7)	0.18 (3.8)	3.71 (5.4)	28.4
LB2	43.1 (8.5)	0.56 (4.8)	0.26 (1.9)	10.3 (7.0)	0.33 (9.0)	<LOD	<LOD	54.6
LB3	19.5 (3.3)	0.94 (7.5)	0.43 (4.9)	10.6 (4.1)	1.59 (3.8)	0.08 (9.4)	1.44 (6.6)	34.4
LB4	19.1 (3.8)	1.23 (6.8)	0.58 (7.8)	10.2 (5.3)	1.32 (6.9)	<LOD	1.11 (5.4)	33.5
LB5	48.2 (6.2)	1.46 (5.2)	0.7 (7.8)	12.1 (5.7)	0.78 (6.7)	0.15 (7.1)	3.71 (5.3)	67.3
LB6	23.1 (2.8)	0.62 (6.5)	0.41 (5.8)	11.2 (4.1)	1.19 (6.0)	0.24 (7.0)	4.12 (5.8)	40.7
LB7 ^c^	34.8 (4.3)	1.65 (2.8)	0.77 (5.9)	10.3 (6.9)	1.68 (4.3)	0.11 (9.3)	4.19 (5.9)	53.4
LB8	49.2 (3.3)	1.43 (6.6)	0.67 (4.6)	12.2 (5.9)	2.17 (6.9)	0.15 (7.0)	7.15 (9.0)	72.9
LC1	25.3 (2.5)	1.23 (4.4)	0.43 (8.0)	9.78 (5.8)	2.59 (6.4)	0.09 (6.1)	2.91 (8.3)	42.1
LC2	21.5 (3.6)	0.98 (2.6)	0.51 (3.7)	10.2 (5.6)	1.60 (5.5)	0.16 (4.3)	4.24 (7.2)	39.2
LC3	35.1 (6.7)	1.11 (3.5)	0.44 (3.6)	8.61 (7.1)	2.36 (5.1)	0.32 (6.4)	9.12 (7.3)	57.0
LC4	33.1 (4.0)	1.25 (3.4)	0.63 (4.9)	10.2 (7.0)	2.61 (4.7)	0.18 (6.7)	5.17 (5.3)	53.1
Mean (mg/g)	30.7	1.07	0.52	10.2	1.58	0.17	4.26	48.1
Fold variation ^b^	3.1	3.9	6.0	1.8	7.8	4.3	7.3	N/A

^a^: Average calculated from *n* = 7 replicates ± % RSD. ^b^: Fold variation = (highest concentration)/(lowest concentration), (<LOD values omitted from this calculation). ^c^: Analytical method validation performed on this sample. ^d^: LB = *L. barbarum*, LC = *L. chinense.*

## Data Availability

The chromatographic files used for PCA analysis and the recovery calculation files used in method validation to support the findings of this study are available from the corresponding author upon request.
